# Ion Binding Properties of a Naturally Occurring Metalloantibody

**DOI:** 10.3390/antib9020010

**Published:** 2020-04-16

**Authors:** Elinaz Farokhi, Jonathan K. Fleming, M. Frank Erasmus, Aaron D. Ward, Yunjin Wu, Maria G. Gutierrez, Jonathan M. Wojciak, Tom Huxford

**Affiliations:** 1 Structural Biochemistry Laboratory, Department of Chemistry & Biochemistry, San Diego State University, 5500 Campanile Dr., San Diego, CA 92182-1030, USA; elinazfarokhi1@gmail.com (E.F.); jon.k.fleming@gmail.com (J.K.F.); frankerasmus@gmail.com (M.F.E.); adurelw@gmail.com (A.D.W.); wuyunjin89@gmail.com (Y.W.);; 2 Apollo Endosurgery, Inc. (formerly Lpath, Inc.) 1120 S. Capital of Tx Hwy, Bldg. 1, Suite 300, Austin, TX 78746, USA; jonwojciak@gmail.com

**Keywords:** antibodies, calcium, germline repertoire, inductively-coupled plasma-mass spectrometry, isothermal titration calorimetry, metalloantibodies, metalloproteins, protein engineering, X-ray crystallography

## Abstract

LT1009 is a humanized version of murine LT1002 IgG1 that employs two bridging Ca^2+^ ions to bind its antigen, the biologically active lipid sphingosine-1-phosphate (S1P). We crystallized and determined the X-ray crystal structure of the LT1009 Fab fragment in 10 mM CaCl_2_ and found that it binds two Ca^2+^ in a manner similar to its antigen-bound state. Flame atomic absorption spectroscopy (FAAS) confirmed that murine LT1002 also binds Ca^2+^ in solution and inductively-coupled plasma-mass spectrometry (ICP-MS) revealed that, although Ca^2+^ is preferred, LT1002 can bind Mg^2+^ and, to much lesser extent, Ba^2+^. Isothermal titration calorimetry (ITC) indicated that LT1002 binds two Ca^2+^ ions endothermically with a measured dissociation constant (*K*_D_) of 171 μM. Protein and genome sequence analyses suggested that LT1002 is representative of a small class of confirmed and potential metalloantibodies and that Ca^2+^ binding is likely encoded for in germline variable chain genes. To test this hypothesis, we engineered, expressed, and purified a Fab fragment consisting of naïve murine germline-encoded light and heavy chain genes from which LT1002 is derived and observed that it binds Ca^2+^ in solution. We propose that LT1002 is representative of a class of naturally occurring metalloantibodies that are evolutionarily conserved across diverse mammalian genomes.

## 1. Introduction

At least one-third, and perhaps as many as one-half, of all functioning proteins are predicted to be metalloproteins [1,2,3]. Included among these metal-dependent biological factors are the vast collection of diverse metalloenzymes, which include many oxidoreductases, proteases, and all protein kinases, as well as metal-dependent extracellular receptors, signaling proteins, transcription factors, and charge transport complexes [4]. However, the involvement of metals in antigen presentation and recognition by proteins of the adaptive immune system remains somewhat of a novelty. In 2009, as part of an effort aimed at developing novel anti-inflammatory and potential anticancer antibody-based therapies that function by selectively binding to signaling lipids, we determined the X-ray crystal structure of the Fab fragment of a humanized mouse monoclonal antibody bound to its antigen sphingosine-1-phosphate (S1P) [5,6]. The complex crystal structure revealed that the humanized anti-S1P antibody, known as LT1009 and derived from the murine anti-S1P antibody LT1002, employs two bridging Ca^2+^ ions that are required for antigen recognition and binding. The two Ca^2+^ are partially coordinated within close proximity (<4 Å) of one another by three aspartic acid residues (Asp30-Asp31-Asp32) from light chain complementarity-determining region loop 1 (CDR-L1) and a fourth aspartic acid (Asp92) from CDR-L3. This arrangement allows for one oxygen atom from the phosphate head group of S1P to complete a *μ* bridging bond to both bound Ca^2+^. Subsequent in vitro analysis confirmed that the bound ions are indeed Ca^2+^ and characterization of the antibody/antigen complex by surface plasmon resonance (SPR) spectroscopy revealed the binding to be extremely favorable with a dissociation equilibrium constant (*K*_D_) in the low nM range. Removal of Ca^2+^, by addition of chelators such as EDTA or EGTA, as well as mutation of Ca^2+^-coordinating amino acid residues completely disrupted the complex to the same or even greater extent than did mutation of residues that contact the antigen directly.

This direct observation of an antibody that was raised by immunization in mice employing interfacial bridging metal ions as a required component of its antigen recognition site called to mind several questions concerning the chemical nature of the anti-S1P antibody:Ca^2+^ interactions. For example, can the antibody bind Ca^2+^ independently of the antigen or is prior interaction with S1P a requirement for Ca^2+^ binding? Does the antibody bind to metal ions other than Ca^2+^? With what affinity is the Ca^2+^ bound? It also raised questions about the biology underlying the anti-S1P antibody such as, did the metalloantibody develop its ability to bind to Ca^2+^ during the processes of somatic recombination and/or affinity maturation or is the potential to coordinate Ca^2+^ evolutionarily conserved within germline antibody gene sequences? In addition, are there other examples of antibodies that employ Ca^2+^ or other metal ions in this fashion to recognize different antigens?

In this study, we report results from a series of structural, in vitro biochemical, and protein engineering experiments that were performed in order to characterize the metal ion binding properties of the anti-S1P antibody and to test the hypothesis that Ca^2+^-mediated antigen binding is an evolutionarily conserved component of a complete and robust antibody repertoire. Our results suggest that the anti-S1P antibody does not require antigen in order to bind Ca^2+^ and that the default Ca^2+^ binding mode is similar to that observed in the LT1009:Ca^2+^:S1P complex X-ray crystal structure. We observe that the antibody binds Ca^2+^ selectively, with an appreciable, but significantly lower capacity for binding Mg^2+^ and even lower affinity toward Ba^2+^. We measure the Ca^2+^ binding affinity and find that its dissociation constant (*K*_D_) is an order of magnitude lower than the typical concentration of Ca^2+^ in plasma, suggesting that the anti-S1P antibody is preloaded with Ca^2+^ in vivo. We identify the Ca^2+^ binding sequence in previously characterized antibodies, some, but not all, of which also require Ca^2+^ for binding to their respective antigens. Finally, we observe that the amino acid sequence signature for Ca^2+^ binding is encoded for within the germline sequences of select antibody kappa light chain gene variable regions across diverse species and that an antibody Fab fragment expressing the mouse germline-encoded sequences from which the anti-S1P antibody is likely derived also binds to Ca^2+^ in solution. These results strongly suggest that the Ca^2+^-dependent antigen binding observed in the anti-S1P antibody is an evolutionarily conserved and diversely applied component of a functional adaptive immune system.

## 2. Materials and Methods

### 2.1. DNA, Oligonucleotides, and Plasmids

Plasmids encoding murine (LT1002) and humanized (LT1009) anti-S1P antibodies have been described previously [5]. For construction of a shuttle plasmid containing both chains of the naïve germline-encoded Fab, we followed the previously reported protocols of Furuta et al. [7]. cDNA encoding the murine Ighv1-78 variable heavy chain in frame with Ighd1-1, Ighj2, and IgG1 constant heavy domain 1 with an N-terminal Gp64 signal peptide and a TEV protease-cleavable C-terminal hexa-histidine tag, was synthesized with codons optimized for insect cell expression and containing 5’-EcoRI and 3’-HindIII sites introduced between the EcoRV sites in a pUC57 plasmid (Genscript). An analogous second pUC57 plasmid was prepared containing a codon-optimized murine light chain gene fragment consisting of Igkv17-121 in frame with Igkj4 and the kappa gene constant light domain with an N-terminal Gp64 signal sequence between 5’-NheI and 3’-SphI restriction sites. Heavy and light chain cDNA were then prepared from the plasmids by double digestion with appropriate restriction enzymes and purified fragments were ligated into the corresponding restriction sites in the pFastBac Dual plasmid (Thermo Fisher). This arrangement places the heavy chain Fab under transcriptional control of the P10 promoter and expression of the light chain under the polyhedrin promoter. DNA sequences for the individual heavy and light chains and for the dual expression pFastBac Dual transfer plasmid are included as Supplementary Materials.

### 2.2. Antibodies and Fab Fragment Generation

Full length murine (LT1002) and humanized (LT1009) anti-S1P antibodies were produced in stable CD-CHO cell lines as previously described [5]. Briefly, antibodies were purified by affinity chromatography on ProSep-vA Ultra resin (Millipore). Fab production from whole IgG has been described previously [6]. Briefly, full length LT1009 IgG was digested at a 100:1 ratio with activated papain (Worthington); the reaction was quenched with iodoacetamide, dialyzed, and purified by anion exchange chromatography. Fab-containing fractions were passed through a ProSep-vA Ultra protein-A column, concentrated to 12 mg/mL, and stored at 4 °C. For preparation of recombinant naïve germline-encoded antibody Fab, we followed protocols for bacmid production, transfection of Sf9 insect cell monolayers, and recombinant baculovirus titer optimization that have been established in this laboratory and reported previously [8]. First, 0.5 L of media was prepared by centrifugation at 500× *g* for 5 min and vacuum filtrated through 0.2 μm nitrocellulose membrane (Millipore) and passed by gravity through 1 mL of Ni Sepharose Fast Flow resin (GE Healthcare) at 4 °C. The column was washed with 25 mM TRIS-HCl pH 8.0, 150 mM NaCl, and 25 mM imidazole, and protein was eluted with wash buffer containing 150 mM imidazole. The eluted protein was treated with 0.1 M EDTA pH 8.0, filtered through a 0.2 μm syringe tip filter (Millipore), and purified on a SuperDex75 16/60 size exclusion chromatography column (GE Healthcare) in 25 mM TRIS-HCl pH 8.0 and 150 mM NaCl. Peak fractions were combined and concentrated to 5 mg/mL in a 10 kDa molecular weight cutoff (MWCO) centrifugal concentrator (Millipore), flash frozen in liquid nitrogen, and stored at −80 °C.

### 2.3. LT1009 Fab:Ca^2+^ Complex Formation and Co-Crystallization

Crystals were grown by the hanging drop-vapor diffusion method at room temperature. Initial screening for conditions that promote crystallization of antigen-free LT1009 Fab were identified by testing Crystal Screen I (Hampton Research) reagents supplemented with 10 mM CaCl_2_. For optimal crystal growth, 1 μL of 12 mg/mL LT1009 Fab was mixed with 1 μL reservoir solution comprised of 0.1 M 4-(2-hydroxyethyl)-1-piperazineethanesulfonic acid (HEPES) pH 7.5, 1.5 M Li_2_SO_4_, and 10 mM CaCl_2_ on a siliconized coverslip and sealed with high vacuum grease over 1 mL of reservoir solution. Plate-like crystals of dimensions 0.3 × 0.3 × 0.02 mm grew for 63 days at room temperature. Crystals were harvested with nylon loops and immersed into 0.1 M TRIS-HCl pH 8.5, 0.2 M Li_2_SO_4_, 30% *w*/*v* polyethylene glycol 4000, 10 mM CaCl_2_, and 10% glycerol for approximately ten seconds prior to flash cooling in liquid nitrogen.

### 2.4. X-ray Crystallography

Synchrotron data were collected at 100 K on a NOIR-1 CCD detector at the Advanced Light Source Beamline 4.2.2, Lawrence Berkeley National Laboratory. Diffraction data indexing and scaling of intensities were carried out with HKL2000 [9]. Data collection statistics are presented in Table 1. Molecular replacement was performed via Phaser-MR in PHENIX using LT1009 Fab (PDB ID 3I9G) as a probe with ions, antigen, and solvent molecules removed, as described previously [10,11]. Rigid-body and initial restrained maximum-likelihood refinements, with all working data to 3 Å resolution, and difference Fourier electron density map calculations to identify potential Ca^2+^ binding sites were carried out using REFMAC5 and FFT, respectively, in CCP4 [12]. Model building was completed in COOT [13]. All further refinements were made in PHENIX [14]. Final assessment of the refined model was carried out using MolProbity [15]. Refinement statistics are presented in Table 1. Final atomic coordinates and structure factors were submitted to the Protein Data Bank (PDB ID: 6VRT). Figures were prepared using PyMOL [16].

### 2.5. Equilibrium Dialysis

Prior to testing, proteins were treated with 0.1 M EDTA pH 8.0 and purified either by dialysis (LT1002/LT3015 FAAS and ICP-MS experiments) or by size exclusion chromatography (naïve germline-encoded Fab antibody ICP-MS). Then, 200 μL of 100 mM stock solution of CaCl_2_·2H_2_O (EMD^®^ #401800) prepared in 20 mM Na-HEPES pH 7.2 and 20 mM NaCl was added to 1.8 mL of 1 mg/mL LT1002, 1 mg/mL LT3015, 1 mg/mL naïve Fab, or Na-HEPES buffer. The resulting solutions at 10 mM CaCl_2_ were applied to 3.5 kDa MWCO dialysis units (Slide-A-Lyzer^TM^ MINI Dialysis Unit #88403). In order to monitor the rate of equilibration, samples were dialyzed against 0.5 L of 20 mM Na-HEPES pH 7.2 and 20 mM NaCl and analyzed at time points 0, 1.5, 3.5, 7.5, and 22.5 h. In subsequent studies, samples were dialyzed against Na-HEPES buffer for 24 h with four changes of buffer. Addition metals were tested by substituting with 100 mM stock solutions of MgCl_2_·6H_2_O (VWR® #VW1483-01), SrCl_2_·6H_2_O (MP Biomedicals #9631 K), BaCl_2_·2H_2_O (EMScience #43129930), Zn(C_2_H_3_O_2_)_2_·2H_2_O (Fisher Scientific® #053087), CdCl_2_ (MD Biomedical #8107K), MnCl_2_·4H_2_O (Fisher Scientific #041398), CoCl_2_·6H_2_O (Fisher Scientific® #040819), NiSO_4_·6H_2_O (EMDTM 35044548), CuSO_4_·5H_2_O (CAS 7758-99-8), YbCl_3_·6H_2_O (CAS 10035-01-05), GdCl_3_ (CAS 10138-52-0), and EuCl_3_·6H_2_O (CAS# 13758-92-7) in 20 mM Na-HEPES pH 7.2 and 20 mM NaCl.

### 2.6. Flame Atomic Absorption Spectroscopy (FAAS)

To begin, 20 μL of sample solution was removed from the dialysis unit, treated nitric acid (5% final concentration), heated at 98 °C for 30 min, and centrifuged at 14,000× *g* for 5 min. Analysis was carried out on a 240 AA instrument (Agilent), which was calibrated by a 6-point dilution of 1.0 ppm CaCO_3_ standard. Instrument settings: lamp current, 70% of 10 mA; compressed air, 9.0 psi; burner height, 15 mm; burner position, 90 °C; fuel flow, 1.1 L/min.; Ca^2+^ absorbance, 422.7 nm; slit width, 0.5 nm. Between samples, the injection port was washed with 5.0% (*v*/*v*) HNO_3_ solution.

### 2.7. Inductively-Coupled Plasma-Mass Spectrometry (ICP-MS)

Samples were prepared as for FAAS and analyzed on an Agilent 4500 Series Inductively Coupled Plasma Mass Spectrometer (ICP-MS) through the Institute for Integrated Research Materials, Environments and Society at California State University, Long Beach.

### 2.8. Isothermal Titration Calorimetry (ITC)

A 20 mM CaCl_2_ solution was titrated at 2.0 μL increments into solutions of 300 μM LT1002, 300 μM LT3015, or no antibody in 50 mM Na-HEPES pH 7.2 and the binding isotherms were measured on a MicroCal™ iTC200 instrument (Malvern) available through Protein Production and Analysis at the Sanford Burnham Prebys Medical Discovery Institute. Data were analyzed by Origin™ software.

### 2.9. Antibody Sequence Analysis

Heavy and light chain variable domain light chain sequences were analyzed on the IgBlast server (http://www.ncbi.nlm.nih.gov/igblast) to identify naïve V, D, and J genes from which the LT1002 anti-S1P antibody is most likely derived [17]. In order to identify antibodies with four Asp residues in CDR-L1 and -L3, a python function was applied to search for three neighboring Asp residues ([D][D][D]) in the abYsis database [18]. Output was analyzed by hand to identify whether the three Asp residues appeared in CDR-L1 and whether a fourth Asp was present in CDR-L3. Identification of conserved kappa light chain gene sequences in species other than mouse was performed by a Blast (http://blast.ncbi.nlm.nih.gov) search with a murine Igkv17-121 gene sequence [19].

## 3. Results

### 3.1. X-ray Crystal Structure of a Ca^2+^-Bound Anti-S1P Antibody Fab Fragment in the Absence of Antigen

**Figure 1:**
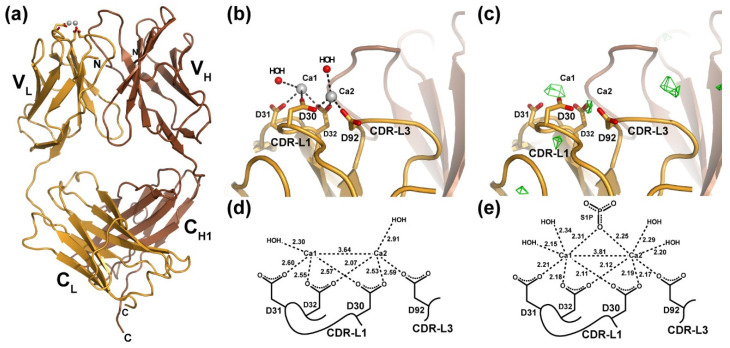
X-ray crystal structure of the LT1009 anti-sphingosine-1-phosphate antibody Fab fragment in complex with Ca^2+^. (**a**) The complete LT1009 Fab fragment is depicted in a ribbon diagram representation with the light chain in gold and the heavy chain in brown. Two Ca^2+^ ions are represented as light grey spheres with the side chains that partially coordinate them shown as sticks. Individual immunoglobulin domains are labeled as are the N- and C-termini of the heavy and light chains. (**b**) A close-up view of the Ca^2+^ binding in LT1009. Side chains from CDR-L1 and CDR-L3 that coordinate Ca^2+^ are depicted as sticks and labeled, and two water molecules that are within generous hydrogen bonding distance of the Ca^2+^ ions are depicted as red spheres. (**c**) An *F*_O_-*F*_C_ difference electron density omit map built with phases from the apo LT1009 molecular replacement probe after rigid-body refinement and contoured at 3.5 sigma (see text) is shown in green and reveals two strong peaks at sites of the bound Ca^2+^ ions. (**d**) A schematic diagram of Ca^2+^ ion coordination by LT1009 with bond lengths in Å and light chain amino acids labeled. (**e**) For comparison, a similar schematic diagram of Ca^2+^ coordination taken from the LT1009:S1P complex X-ray crystal structure is shown.

We first analyzed whether the anti-S1P antibody is capable of binding Ca^2+^ in the absence of S1P or, alternatively, if their interaction is dependent upon the presence of antigen (Figure 1). We also wished to determine by what mode the antibody might bind Ca^2+^ in the absence of antigen. This second question arose from our analysis of the results from a previous study in which X-ray crystallography was employed to determine the site of Ca^2+^ binding by the murine Q425 antibody. Q425 selectively binds to the extracellular portion of the human CD4 co-receptor in a Ca^2+^-dependent manner that inhibits steps subsequent to virus binding and protects against HIV infection of CD4^+^ T cells [20]. By crystallizing and determining the X-ray crystal structure of the Q425 Fab fragment separately in the presence of 10 mM Ba^2+^, 10 mM Ca^2+^, and 10 mM EDTA, researchers concluded that one Ca^2+^ binds to a site at the interface between the light and heavy chains, employing amino acid side chains from CDR-H3, CDR-L2, and -L3 [21]. This site, which was also able to accommodate binding of the significantly larger Ba^2+^ ion, is completely different than what we observed in the LT1009:Ca^2+^:S1P complex structure, where two Ca^2+^ are coordinated by four Asp residues from light chain CDR-L1 and -L3 and the phosphate group on the S1P antigen [6].

Following closely the approach employed in the Q425 structural study, we prepared crystals of the LT1009 humanized anti-S1P antibody Fab fragment in 10 mM Ca^2+^ and collected a complete set of X-ray diffraction data. Experimental estimates of phase were provided by molecular replacement with a search model consisting of atomic coordinates for LT1009 from the Fab:Ca^2+^:S1P complex crystal structure (PDB ID 3I9G) with S1P antigen and all nonbonded atoms (water, ions) removed. After rigid-body and restrained refinement of the model against diffraction data to 3.0 Å resolution, *F*_O_-*F*_C_ difference Fourier maps were calculated and analyzed for the presence of unaccounted for peaks of electron density. Two spheres of positive electron density were visible at the CDR-L1/CDR-L3 binding sites at a contour level of 3.5 σ (Figure 1c). These corresponded in their positions almost exactly to the sites of the two Ca^2+^ observed in the antigen-bound LT1009 Fab complex X-ray crystal structure. Importantly, no significant peak was observed at the interface between LT1009 Fab heavy and light chain variable domains, strongly suggesting that, at 10 mM concentration, Ca^2+^ is found at the same sites as was observed in the antigen-bound anti-S1P complex crystal structure and not in the site proposed by crystallographic analysis of antibody Q425. The significance of this observation is that the light chains of Q425 and LT1009 are highly similar with CDR loop sequences that differ at only four positions and preservation of all four Ca^2+^-coordinating Asp residues.

We continued with model building and refinement against all data to a resolution limit of 2.56 Å, ultimately yielding a model for LT1009 Fab in complex with Ca^2+^ with a working *R*-factor (*R*_work_) of 20.9% and *R*_free_ of 26.0% and with very good stereochemistry (Table 1). The crystallographic model, which consists of heavy chain amino acids 1-217, light chain amino acids 1-214, two Ca^2+^, 28 waters, and one sulfate ion, reveals the familiar and expected Fab fragment immunoglobulin domain organization (Figure 1a). The antigen-free and -bound Fab models are practically identical with a root-mean squared deviation (rmsd) of 0.630 Å for C_α_ positions upon superposition. The elbow angles, a measure for relative displacement of the variable and constant domains upon antigen binding, are also in close agreement with the elbow angle for the Fab:Ca^2+^:S1P complex structure at 167.5° and that of the antigen-free Fab:Ca^2+^ complex measuring 170.1° [22]. Where the two crystallographic models differ is with respect to the average distance in the bonds to the two Ca^2+^ ions. The interionic distance of 3.64 Å in the antigen-free model compares well with that of the antigen-bound complex, where the refined distance between the bound Ca^2+^ ions is 3.81 Å (Figure 1d,e). This suggests that the cationic charges are effectively shielded from one another upon antibody binding. However, each of the six bonds between the Asp side chain oxygen atoms and Ca^2+^ as well as to two ordered water molecules are longer than the corresponding bonds in the antigen-bound complex. Although the magnitude of these differences is less than the estimated coordinate error as calculated by maximum-likelihood methods, which for the antigen-free model is 0.51 Å, the resulting longer bond distances agree well with established Ca–O bond distances while the significantly shorter Ca–O bonds observed in the 1.9 Å resolution antigen-bound complex approach the limits for what has been observed [23]. Consistent with this interpretation, we note that the refined *B*-factors associated with the two bound Ca^2+^ ions are higher on average than for protein or solvent atoms throughout the model (Table 1). Therefore, our crystallographic analyses suggest that at 10 mM Ca^2+^ concentration and in the absence of S1P antigen, two Ca^2+^ bind to the anti-S1P antibody through association with the four Asp side chains from CDR-L1 and -L3 and that binding of the phosphate head group of S1P is accompanied by Ca–O bond shortening that likely correlates with significant increased Ca^2+^ binding affinity.

### 3.2. Detection of Ca^2+^ Binding by LT1002 in Solution

**Figure 2:**
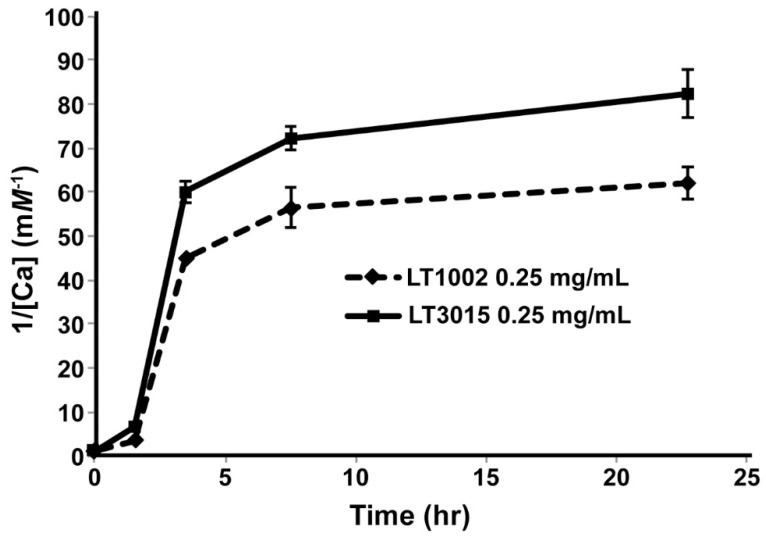
The rate of decrease in Ca^2+^ concentration versus time as monitored by FAAS after equilibrium dialysis. The Ca^2+^ concentrations (reported in reciprocal units to aid in visualization) remaining in solutions of 10 mM CaCl_2_ and either 0.25 mg/mL LT1002 anti-S1P antibody (dashed line) or control LT3105 anti-LPA antibody (solid line) were measured via FAAS at 0, 1.5, 3.5, 7.5, and 22.5 h of dialysis against excess buffer.

Having established by X-ray crystallography that the anti-S1P antibody binds two Ca^2+^ at the same sites as was previously observed in the LT1009 Fab:Ca^2+^:S1P complex crystal structure, we next wished to establish methods for assessing Ca^2+^ binding in solution. To this end, we employed equilibrium dialysis of whole murine LT1002 anti-S1P IgG1 antibody and detection by flame atomic absorption spectroscopy (FAAS). By this approach, buffered solutions of LT1002 anti-S1P antibody that had been rendered metal ion free by pretreatment with EDTA were incubated in solution containing Ca^2+^ and dialyzed against excess amounts of Ca^2+^-free buffer. In order to monitor the rate of dialysis under the conditions tested, we first carried out an experiment in which the amount of Ca^2+^ retained within the dialysate fraction was monitored over time relative to similarly prepared and buffered whole LT3015, an antibody specific for lysophosphatidic acid (LPA) that we had previously demonstrated does not involve Ca^2+^ in binding to its lipid antigen [24]. By this approach, LT1002 anti-S1P antibody-dependent retention of Ca^2+^ could be detected relative to LT3015 anti-LPA antibody by FAAS after 24 h, and the amount of Ca^2+^ remaining within the dialyzed antibody samples did not change significantly during the final 15 h of dialysis (Figure 2).

**Figure 3:**
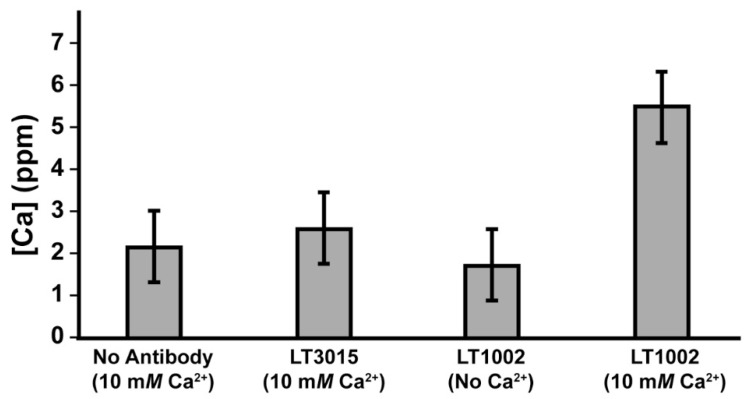
Comparison of Ca^2+^ concentration measured by FAAS after 24 h dialysis of 10 mM Ca^2+^ in the absence of antibody, in the presence of 0.9 mg/mL LT3015 anti-LPA antibody, and in the presence of 0.9 mg/mL LT1002 anti-S1P antibody.

We next performed an experiment in which the concentration of Ca^2+^ remaining after dialysis was measured by FAAS, and comparison was made between samples containing 10 mM CaCl_2_ in 20 mM Na-HEPES pH 7.2 buffer without antibody, 10 mM CaCl_2_ in buffer with 0.9 mg/mL LT3015, 0.9 mg/mL LT1002 in buffer without Ca^2+^, and 10 mM CaCl_2_ in buffer with 0.9 mg/mL LT1002 (Figure 3). After 24 h, the samples containing LT1002 and 10 mM CaCl_2_ were observed to contain significantly higher amounts of calcium on average. These experiments strongly suggest that the anti-S1P antibody is capable of binding Ca^2+^ in solution independent of its antigen.

### 3.3. Metal Ion Binding Specificity of LT1002

We next wished to determine whether metal ions other than Ca^2+^ are bound efficiently by LT1002 in solution. For this experiment, we again employed replicate measurements of metal ions in solution after equilibrium dialysis in the presence of buffer alone, with LT1002 anti-S1P antibody, or in the presence of control LT3015 anti-LPA antibody. In order to both improve the sensitivity and accuracy of our metal ion detection as well as expand the repertoire of metals we could test, we employed inductively-coupled plasma-mass spectrometry (ICP-MS) detection.

**Figure 4:**
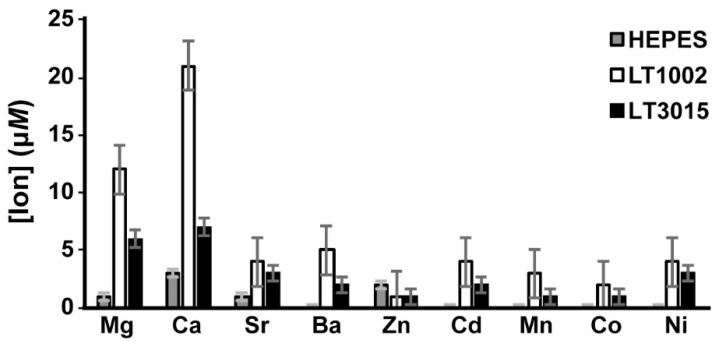
Average metal ion concentrations after 24 h of dialysis in the presence of Na-HEPES buffer alone (grey), LT1002 anti-S1P antibody (white), or control LT3015 anti-LPA antibody. Triplicate samples were analyzed by ICP-MS.

As observed previously in the FAAS experiments, LT1002 retains Ca^2+^ to a significantly greater degree than does the control LT3015 antibody (Figure 4). Interestingly, we also observed by this approach that LT1002 can bind to Mg^2+^, though to a significantly lower extent than it binds to Ca^2+^. It bears mentioning that the original LT1009 anti-S1P antibody Fab:Ca^2+^:S1P complex crystal was grown in 100 mM MgSO_4_ and that it was initially assumed that the bridging metals in the X-ray crystal structure must be Mg^2+^ before crystallographic model refinement suggested otherwise. The presence of Ca^2+^ in the complexes was then confirmed by ICP spectroscopy [6]. Taken together these observations suggest that the anti-S1P antibody binds appreciably to Mg^2+^ in solution, though it displays a preference for Ca^2+^ when binding to its S1P antigen. This is likely a consequence of the difference in ionic radius of Mg^2+^ and Ca^2+^ and the constraints of coordinating carboxylate groups of three vicinal aspartic acid residues in CDR-L1.

Interestingly, we observed a slight, but significant, increased binding of Ba^2+^ by LT1002 relative to the LT3015 control antibody. As mentioned previously, the Ca^2+^-dependent anti-CD4 antibody Q425 was observed by X-ray crystallography to bind Ba^2+^ at a distinct site at the interface between its variable heavy and light domains. This same site was also observed to house one Ca^2+^ when the Q425 Fab fragment was crystallized in 10 mM CaCl_2_, though the coordination geometry, bond lengths, and ligand atoms are nonideal [21]. It is possible that Ba^2+^ could bind in a similar manner in LT1002.

We did not observe significant binding to Sr^2+^. Similarly, we did not observe binding by LT1002 anti-S1P antibody to any of the transition metal ions tested including Zn^2+^, Cd^2+^, Mn^2+^, Co^2+^, and Ni^2+^. Several of these ions require rather specific ligand atom types and bonding geometries, whereas Ca^2+^ has been shown to be capable of adapting to a much broader range of coordination spheres [25]. Finally, in single experiments we failed to observe any evidence of LT1002 binding with lanthanide metal ions Yb^3+^, Gd^3+^, or Eu^3+^ (data not shown).

### 3.4. Isothermal Titration Calorimetry of Ca^2+^ Binding to LT1002

In order to thermodynamically characterize the interaction of Ca^2+^ and the anti-S1P antibody in solution, we next carried out isothermal titration calorimetry (ITC). By this approach, controlled amounts of Ca^2+^ were introduced to a HEPES buffered solution of LT1002 antibody and the change in electrical current required to maintain a steady solution temperature was carefully measured. This allows for direct determination of the enthalpy associated with binding. Analysis of the titration endpoint can be fit to determine binding association constants, through which enthalpic components of the binding free energy can be deduced. Through this method, full thermodynamic profiles of metals for proteins, including binding affinities, have been determined [26,27].

**Figure 5:**
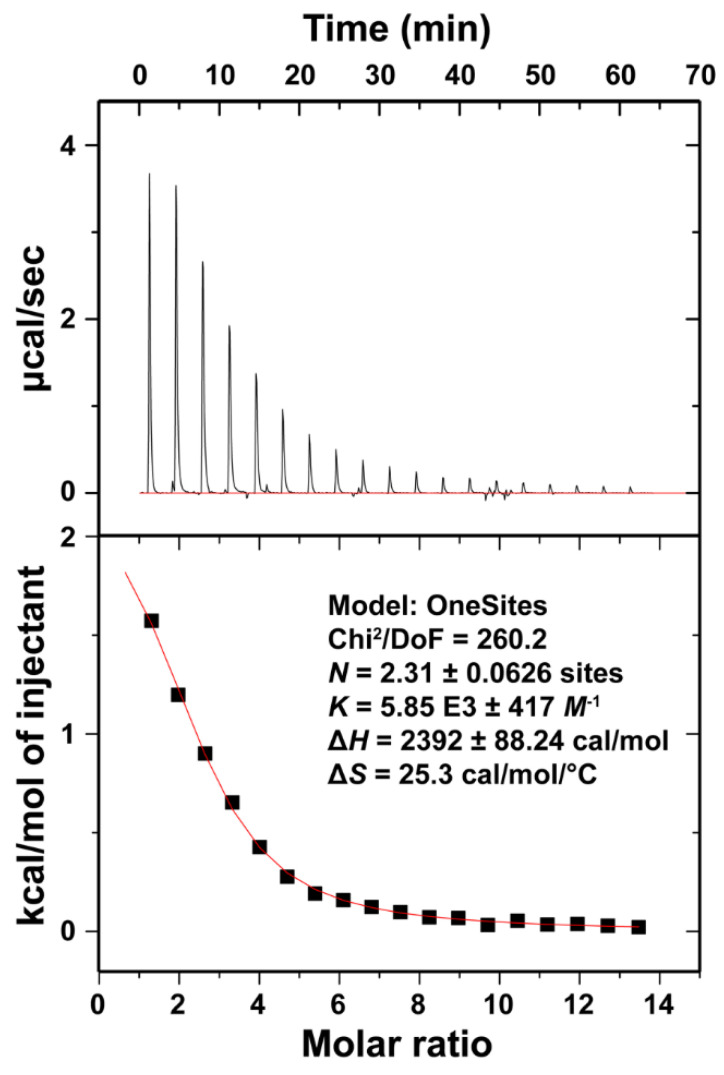
Isothermal titration calorimetry (ITC) analysis of Ca^2+^ binding by the LT1002 anti-S1P antibody. Binding isotherms were measured as CaCl_2_ was titrated into a Na-HEPES buffered solution containing LT1002. The data fit to a model in which slightly more than 2 ions bind per site with an equilibrium dissociation constant (*K*_D_) of 171 μM.

ITC measurements on the LT1002 antibody interaction with Ca^2+^ immediately revealed that binding of the metal is an endothermic (heat requiring) process (Figure 5). This is interesting as it implies that the driving force behind assembly of the observed antibody/metal complex is an increase in entropy (disorder) in the complex relative to the free protein and ions in solution. The increased disorder that accompanies complex formation is almost certainly a consequence of freeing waters from the hydration sphere of Ca^2+^ upon binding to LT1002. Indeed, a similar dependence upon increased entropy was observed when Ca^2+^ was bound by EDTA in Na-HEPES buffered solution, though the binding affinity was several orders of magnitude tighter for EDTA:Ca^2+^ than we measured for LT1002:Ca^2+^ complex formation [28].

The titration data were fit starting from a model of two ions per binding event and refined to roughly 2.3 ions binding with an average equilibrium binding affinity of 171 μM (*K*_D_). This represents a relatively low affinity interaction. However, when one considers the energy associated with shielding the charges on the two Ca^2+^ as they bind within close proximity of one another as well as the fact that only three coordination sites on each of the two bound ions are filled by the carboxylate oxygens of the critical four Asp residues, it seems reasonable that the binding affinity would not be too strong. The measured value is certainly consistent with our observation that at 10 mM CaCl_2_, there appears to be full occupancy of Ca^2+^ bound throughout the crystal. From the perspective of physiological relevance, 171 μM is roughly an order of magnitude lower than the typical plasma concentration of Ca^2+^. This suggests that under physiological conditions, the anti-S1P antibody exists in it predominantly Ca^2+^-bound state. As indicated from our previous antigen binding and X-ray crystallographic studies, subsequent binding to S1P antigen significantly increases the Ca^2+^ binding affinity. Titration of Ca^2+^ into buffered LT3015 anti-LPA antibody or HEPES buffer alone revealed no interaction between Ca^2+^ and HEPES or the control antibody (Figure S1). Finally, measurement of LT1002:Ca^2+^ binding was performed three times with similar results.

### 3.5. Sequence Conservation of LT1002 Ca^2+^-Coordinating Residues in Other Antibodies

Upon confirming that LT1002 binds Ca^2+^ in solution and having previously identified the specific amino acid residues responsible for Ca^2+^ binding, we next sought to determine what previously characterized antibodies might also contain these residues. To this end, we performed a search through the abYsis database for antibodies with three neighboring Asp residues (corresponding to Asp30-Asp31-Asp32) in CDR-L1 and a fourth Asp residue equivalent to Asp92 in CDR-L3 [18]. Among the antibodies that fit this profile is the murine Q425 anti-CD4 antibody that has been described previously (Table 2). Q425 was shown by surface plasmon resonance (SPR) spectroscopy to bind to its CD4 antigen with 85 μM affinity (*K*_D_) in the absence of Ca^2+^ and 1.6 nM affinity in the presence of 25 mM Ca^2+^, clearly identifying it as a bona fide metalloantibody [21]. It is worth noting that when the researchers employed SPR to assess the affinity of Ca^2+^ for Q425 indirectly, they arrived at a value of 187 μM, which agrees surprisingly well with what we measured by ITC for Ca^2+^ binding to LT1002. This is despite the fact that X-ray crystallography of the Q425 Fab fragment in 10 mM CaCl_2_ suggests that only one Ca^2+^ binds to Q425 at a site that differs completely from what is observed in the anti-S1P antibody crystal structures [21].

A second antibody identified by virtue of its close homology to LT1002 that includes each of the four Ca^2+^-coordinating Asp residues is murine 2C10, which was originally identified as an anti-double-stranded DNA (anti-dsDNA) antibody expressed in an MRL/1 mouse model of the autoimmune disease systemic lupus erythematosus (SLE) [29]. Subsequent studies on 2C10 have identified its preference for DNA containing dA:dT base pairs over dC:dG and enhancement of DNA oxidative cleavage [30]. Recently, it was shown that 2C10:dsDNA complexes can enter live monocytes in culture and promote the expression of cytokines associated with SLE [31]. No direct evidence for the involvement of divalent metal cations has ever been reported for 2C10, though the potential for metal-mediated coordination to phosphodiesters of the DNA backbone is an intriguing possibility.

A third antibody that shares all of the LT1002 Ca^2+^-coordinating amino acid residues is the MR1 antibody that binds to a polypeptide epitope near the N-terminus of a mutant epidermal growth factor receptor (EGFRvIII). X-ray crystallography of the complex between an MR1 single chain variable domain (scFv) and a 13-mer antigen peptide reveals that none of the CDR-L1 amino acids are involved in antigen binding and that no metal ions are observed or suspected in the complex [32]. The CDR-L1 loop of the MR1 scFv adopts a conformation that is extremely similar to that observed in the anti-S1P and Q425 X-ray crystal structures, and neither it nor CDR-L3 contact the antigen directly. Therefore, it is possible that metal binding by the MR1 light chain might serve to enhance the interaction with an even larger epitope. However, a simpler alternative explanation is that antibodies bearing amino acids with Ca^2+^-binding potential do not necessarily require metals for antigen recognition and binding.

### 3.6. LT1002 Ca^2+^-Coordinating Residues Are Encoded in Diverse Light Chain Germline Sequences

One of the fundamental questions that arose in response to our observation of the critical role played by Ca^2+^ in antigen binding by LT1002 is in regard to the manner by which the anti-S1P antibody developed its ability to bind Ca^2+^. Individual antibodies arise in developing B lymphocytes through a series of well characterized yet complicated molecular biological reactions that include somatic hypermutation and recombination of naïve germline-encoded starting sequences throughout the processes of B cell receptor (BCR) gene assembly and antibody affinity maturation [33]. We wished to determine whether the potential for Ca^2+^ binding developed during these processes or if it can be found encoded within the germline DNA sequences of antibody light chain genes. Lacking access to the cells in which the anti-S1P antibody originally developed, we relied upon analysis by the IgBlast server to identify the likely murine variable and joining genes from which LT1002 is derived [17]. This returned the variable kappa light chain gene Igkv17-121 as a 96.8% likely source for the LT1002 light chain variable domain [34]. Analysis of Igkv17-121 reveals that only nine base mutations differ between what is encoded by the gene and the LT1002 light chain. Of these, five missense mutations alter the identities of amino acids. Ser26 encoded for by the germline sequence changes Thr in LT1002: Tyr36 becomes Phe, Lys45 is altered to Asn, Thr53 changes to Ile, and Val72 becomes Leu. Notably, the four critical Ca^2+^-coordinating residues, Asp30, Asp31, Asp32, and Asp92, are present in the naïve germline variable gene sequence (Table 2). IgBlast identifies Igkj4 as the source for the light chain joining (J) gene. The heavy chain was identified as derived from in-frame junctions of Ighv1-78 (V), Ighd1-1 (D), and Ighj2 (J).

Even more intriguingly, the mouse immunoglobulin kappa gene cluster contains a second, highly conserved variable domain sequence, Igkv17-127, that also encodes for the four Ca^2+^-coordinating Asp residues of LT1002 [35]. Additionally, the unexpressed variable light chain pseudogene Igkv17-134 bears close homology to the consensus Ca^2+^-binding motifs (Table 2). The observation that the Ca^2+^-coordinating Asp residues are encoded within the germline sequences of two murine variable light chain genes inspired us to seek out similar patterns within other mammalian genomes. Database searches revealed individual genes in each of rat (Igkv17S1), macaque (Vk5.4), and human (Igkv5-2) genomes that bear each of the four Asp residues in positions that correspond to murine LT1002 (Table 2). Tellingly, the translated amino acid sequences flanking the four Asp residues as well as in CDR-L2 are also highly conserved, indicating that these residues play important roles and strongly suggesting that this germline sequence encoding for antibody CDR loops that we have observed to involve bridging metal ions in antibody binding is evolutionarily conserved across diverse mammalian genomes.

### 3.7. The Germline-Encoded “Precursor” to LT1002 Binds Ca^2+^

**Figure 6:**
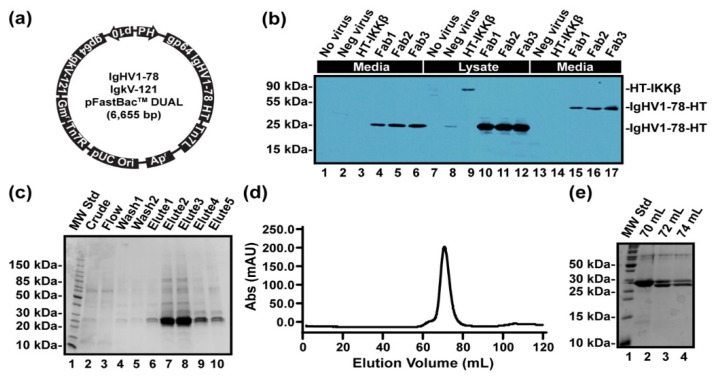
Expression and purification of LT1002 germline-encoded precursor Fab fragments from Sf9 insect cell suspension cultures. (**a**) pFastBac Dual plasmid with both heavy and light chain inserted and polyhedrin promoter for the heavy chain and p10 promoter for the light chain was designed. Signal peptide gp64 was used to secret the antibody. (**b**) Anti-His Western blot detection of secreted metalloantibody precursor Fab fragment from P1 baculovirus-infected Sf9 cells. 1, 2, and 3 secreted Fab antibodies, 4. negative control (cells with no virus), 5. negative control (intact virus), 6. IKK (Intracellular protein control), 7, 8, and 9 intracellular Fab antibodies, 10, 11, and 12 negative control, 13,14, and 15 secreted non-denaturing Fab antibodies. (**c**) Coomassie-stained 15% SDS PAGE gel for samples from Ni column purification. The size of the Fab antibody was 24kD, and the E2-E4 elutes contained most of the combined proteins. The double bands show heavy and light chains of the antibody. (**d**) Size exclusion chromatography using Superdex 75 16/60 of the His-tagged Fab antibody. (**e**) Coomassie-stained 15% SDS PAGE gel indicating the fractions from SEC column purification.

In order to test the hypothesis that binding to Ca^2+^ is preserved within germline sequences of antibody light chain genes across diverse mammalian species, we expressed and purified recombinant Fab fragments of the naïve germline-encoded light and heavy chain gene precursors to the murine LT1002 anti-S1P antibody (Figure 6). Using the approach developed by Furuta et al., we engineered recombinant baculovirus containing the germline-encoded naïve sequences for LT1002 light and heavy chains with N-terminal Gp64 signal peptides on each chain and a C-terminal hexahistidine tag on the heavy chain [7]. Our version of the Igkv17-121 light chain gene contained an in-frame Igkj4 and constant light chain. The DNA sequences used are available as Supplementary Materials. Expression of the antibody in Sf9 insect cell suspensions and Western blot detection with an anti-His antibody revealed that significant amounts of the heavy chain were secreted into media and that the size of the heavy chain band correlated to intact, covalently Cys-linked Fab under nonreducing conditions (Figure 6b). After optimizing virus titer for optimal expression yield, antibody Fab fragments were purified to homogeneity via nickel affinity and size exclusion chromatography (Figure 6c–e).

**Figure 7:**
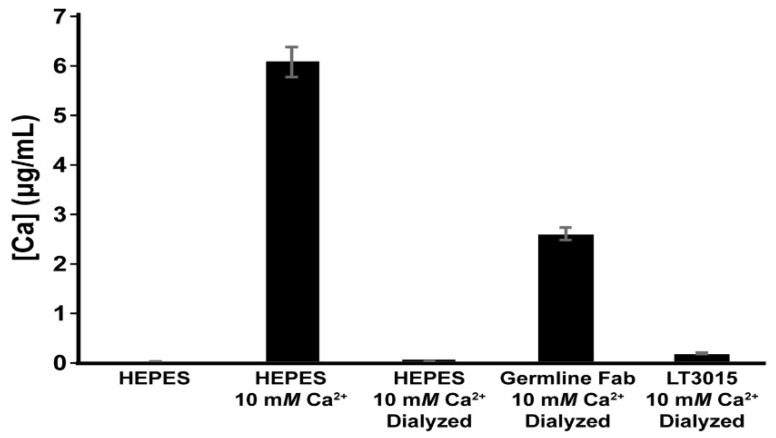
ICP-MS detection of Ca^2+^ from aqueous samples. “HEPES” and “HEPES 10 mM Ca^2+^” are samples that were not subjected to dialysis and represent the low and high levels of Ca2+, respectively, used in the solutions during this experiment. Samples labeled Dialyzed were measured after dialysis against excess Na-HEPES pH 7.2 buffer.

The purified naïve germline antibody Fab was tested for its ability to bind Ca^2+^ by equilibrium dialysis and ICP-MS. For this experiment, solutions of Na-HEPES buffer at pH 7.2 with and without the addition of 10 mM CaCl_2_ were used as controls to establish the detection range of the ICP-MS instrument (Figure 7). After extensive dialysis of the buffered 10 mM CaCl_2_ solution, there was almost no Ca^2+^ detected. Incubation of 10 mM CaCl_2_ in the presence of the germline-encoded Fab fragment, however, resulted in retention of a significantly increased amount of Ca^2+^. Measurably significant levels of Ca^2+^ were detected after dialysis in the presence of control LT3015 anti-LPA antibody, but these were only slightly higher than for the buffer control. We conclude that the CDR amino acid sequences encoded for by the light chain of murine kappa light chain gene Igkv17-121 and conserved across genomes of diverse mammals harbor inherent Ca^2+^ binding potential.

## 4. Discussion

The incorporation of metals as interfacial bridging factors greatly expands the potential of proteins for folding stability, catalysis, and selective binding to molecular targets. Although numerous metalloproteins have been identified and characterized, the involvement of interfacial metals in antigen recognition by proteins of the adaptive immune system has remained somewhat dubious. Where there is clear evidence for the involvement of metals in antigen binding, as in the case of CD4 binding by the murine Q425 antibody, it is unclear exactly how Ca^2+^ binding promotes the observed significant enhancement of antigen binding and resultant disruption of HIV infection [21]. Another instance of suspected involvement of bridging metal ions in immunocomplex formation involves the human T Cell Receptor (TCR) ANi2.3 that is associated with Ni^2+^-contact hypersensitivity [36,37]. Previous structural and biochemical characterization of a complex between TCR ANi2.3 and MHCII protein DR52c bearing a peptide that was identified by in vitro selection to bind with high affinity and activate the ANi2.3 T cell reveals that the ε-amino group from a particular Lys residue on the peptide antigen interacts with TCR residues that likely provide a binding site for a bridging Ni^2+^ [38]. However, direct observation of the bound Ni^2+^ has not been reported. Our structural and biochemical analyses of the murine LT1002 anti-S1P antibody and its humanized form, LT1009, provide a definitive case for the involvement of Ca^2+^ as a bridging factor to aid in selective binding to a target antigen.

Our X-ray crystallographic data strongly suggest that at 10 mM Ca^2+^ concentration and in the absence of S1P antigen, two Ca^2+^ bind at the same site as was observed in the antigen-bound complex. The two Ca^2+^ directly contact Asp30, Asp31, and Asp32 from CDR-L1 as well as Asp92 from CDR-L3 through coordinate bonds that are within the expected range for Ca–O [23]. This arrangement leaves three sites available (assuming octahedral coordination) for each bound Ca^2+^ that can be used to bind antigens. It bears mentioning that this portion of the anti-S1P antibody is exposed to solvent within the crystal and that no atoms from neighboring complexes are involved in stabilizing the two bound Ca^2+^. We conclude that this is the preferred mode for Ca^2+^ binding to the anti-S1P antibody and that the presence of antigen, while stabilizing the interaction and shortening the distances between bonded atoms, does not significantly alter the Ca^2+^ binding site. This is an important conclusion as comparison with the antigen-free Q425:Ca^2+^ complex X-ray crystal structure reveals an alternative Ca^2+^ binding site [21]. This is despite the fact that the Q425 and LT1002 antibodies display 94.7% sequence identity within their variable domains including sharing all of the LT1002 Ca^2+^-coordinating residues. In the absence of a more detailed study, it is impossible to know for certain how Q425 employs Ca^2+^ to bind to its CD4 antigen. Nonetheless, at present, it appears that antibody kappa light chains derived from the murine Igkv-17 might be capable of employing multiple modes of metal binding in order to recognize and bind to diverse antigens.

Our characterization of ion binding by LT1002 in solution reveals that Ca^2+^ is preferred, though Mg^2+^ can also associate with the anti-S1P antibody. Ca^2+^ and Mg^2+^ bind to similar sites on proteins although, due primarily to the greater ionic radius of Ca^2+^ and consequent exclusive requirement for monodentate ligand binding, with differing consequences. In several notable cases, Ca^2+^ binding impedes catalysis by metalloenzymes where the smaller Mg^2+^ co-factor favors it [39,40]. ITC revealed a relatively low Ca^2+^ binding affinity for LT1002 with a measured dissociation constant (*K*_D_) for binding to two Ca^2+^ at 171 μM. This is significant on several counts. First, this is roughly ten times lower than the typical concentration of Ca^2+^ in blood plasma, which suggests that LT1002 is generally preloaded with Ca^2+^ as it circulates in the blood stream. We had previously speculated that the antibody might bind to Ca^2+^ and S1P simultaneously through a concerted binding mechanism or that initial antigen binding might induce subsequent affinity for Ca^2+^. In light of the present study, we are confident that circulating anti-S1P antibodies associate with Ca^2+^ independent of antigen. Second, the low affinity correlates with the significant increase in bond distances to the two Ca^2+^ ions observed in the antigen-free X-ray crystal structure relative to its S1P antigen-bound structure. This suggests that the Ca^2+^ are bound with significantly higher affinity upon antigen binding and explains why Ca^2+^ remained bound even at 100 mM Mg^2+^ concentration used for crystallization of the S1P antigen-bound complex [6]. Finally, our measured value for Ca^2+^ binding agrees remarkably well with that deduced in an elegant SPR study on the closely related anti-CD4 Q425 antibody, which also showed that Ca^2+^ affinity was increased by a factor of greater than 50,000 in the presence of its CD4 protein antigen [21].

Identification of the interfacial Ca^2+^-coordination signature (three neighboring Asp residues at positions 30–32 in CDR-L1, Asp at position 92 in CDR-L3) in other antibodies immediately raises the question of whether implementation of bridging Ca^2+^ ions is a common mechanism employed by antibodies. Other than Q425, neither of the other two antibodies identified as containing the Ca^2+^-coordination signature, the 2C10 anti-dsDNA antibody, and the MR1 anti-EGFRvIII antibody are known or suspected to rely upon bridging metal ions to bind their respective antigens. By expressing the murine Igkv17-121 germline sequence as part of a naïve antibody Fab fragment and confirming its ability to bind Ca^2+^ in solution, we have proven that Ca^2+^ binding potential is inherent to these sequences and preserved within the mouse germline repertoire. Our observation of antibody light chain genes that contain the Ca^2+^-coordinating residues within germline sequences of diverse mammals makes a strong case for interfacial Ca^2+^-dependent antigen recognition as an evolutionarily conserved component of the mammalian antibody repertoire.

## Figures and Tables

**Table 1:** X-ray crystallography data collection and refinement statistics.

	LT1009 Fab:Ca^2+^ Complex
Data collection	
X-ray source	ALS 4.2.2
Wavelength (Å)	1.0000
Space Group	*I*222
Unit cell (Å)	
a	87.80
b	114.41
c	133.70
Molecules/asymm. unit	1
Resolution Range (Å) ^1^	50.0–2.55 (2.59–2.55)
*R*_sym_ (%)	8.7 (77.0)
Observations	161,860
Unique reflections	22,135
Completeness (%)	100.0 (100.0)
Redundancy	7.3 (5.8)
<*I*/σ>	23.6 (2.3)
Refinement	
Number of reflections	22,124
*R*_work_ (%)	20.9 (35.3)
*R*_free_^2^	26.0 (46.4)
Protein atoms	3365
Ca^2+^/H_2_O/SO_4_^2−^ atoms	42
Geometry (R.m.s.d.)	
Bond lengths (Å)	0.012
Bond angles (°)	1.142
Mean *B* (Å ^2^)	
Protein	60.5
Solvent	51.5
Calcium ions	93.6
Ramachandran plot ^3^	
Favored	93.6
Allowed	6.2
Disallowed	0.2
MolProbity score ^4^	1.81
PDB accession code	6VRT

^1^ Data in parentheses are for highest resolution shell. ^2^ Calculated against a cross-validation set of 5.1% of data selected at random prior to refinement. ^3^ Calculated by MolProbity [15]. ^4^ Combines clashscore, rotamer and Ramachandran evaluations to a single score, normalized to the same scale as X-ray resolution [15].

**Table 2:** Conservation of LT1002 metal-contacting residues (bold) in CDR-L1 and -L3.

Antibody	CDR-L1	CDR-L2	CDR-L3	Organism	Antigen	Metal
LT1002	ITTTDI**DDD**MN	EGNILRP	LQS**D**NLPFT	Mouse	S1P	Ca^2+^
Q425	ITSTDI**DDD**MN	EGNTLRP	LQS**D**TLPLT	Mouse	CD4	Ca^2+^/Ba^2+^
2C10	ITNTDI**DDD**MN	EGNTLRP	LQS**D**NMPLT	Mouse	dsDNA	
EGFRvIII	ITSTDI**DDD**MN	EGNTLRP	LQS**D**NLPLF	Mouse	EGFR	
Gene	CDR-L1	CDR-L2	CDR-L3	Organism		
Igkv17-121	ITSTDI**DDD**MN	EGNTLRP	LQS**D**NLP..	Mouse		
Igkv17-127	ITSTDI**DDD**MN	EGNTLRP	LQS**D**NMP..	Mouse		
Igkv17-134 ^1^	THNTDI**DDE**MH	EGNTLHP	LQS**G**NMP..	Mouse		
Igkv5-2	KASQDI**DDD**MN	EATTLVP	LQH**D**NFP..	Human		
Igkv17S1	KTSTDI**DDD**MN	EGNTLRP	QQS**D**NVP..	Rat		
Vk5.4	RAGQDI**DDD**MN	DATTLVS	LQH**D**NFP..	Macaque		

^1^ Igkv17-134 is a pseudogene.

## Supplementary Materials

The following are available online at https://www.mdpi.com/2073-4468/9/2/10/s1, Supplementary Figure S1: DNA sequences employed in production of naïve germline-encoded metalloantibody.
