# Supplementary material for: Ion Binding Properties of a Naturally Occurring Metalloantibody
Source: Antibodies (Basel). 2020 Apr 16;9(2):10. doi: 10.3390/antib9020010 (PMC7345679; doi:10.3390/antib9020010)
Supplement: Supplementary file 1 [file antibodies-09-00010-s001.pdf]

SUPPLEMENTARY MATERIAL

Supplementary Figure S1

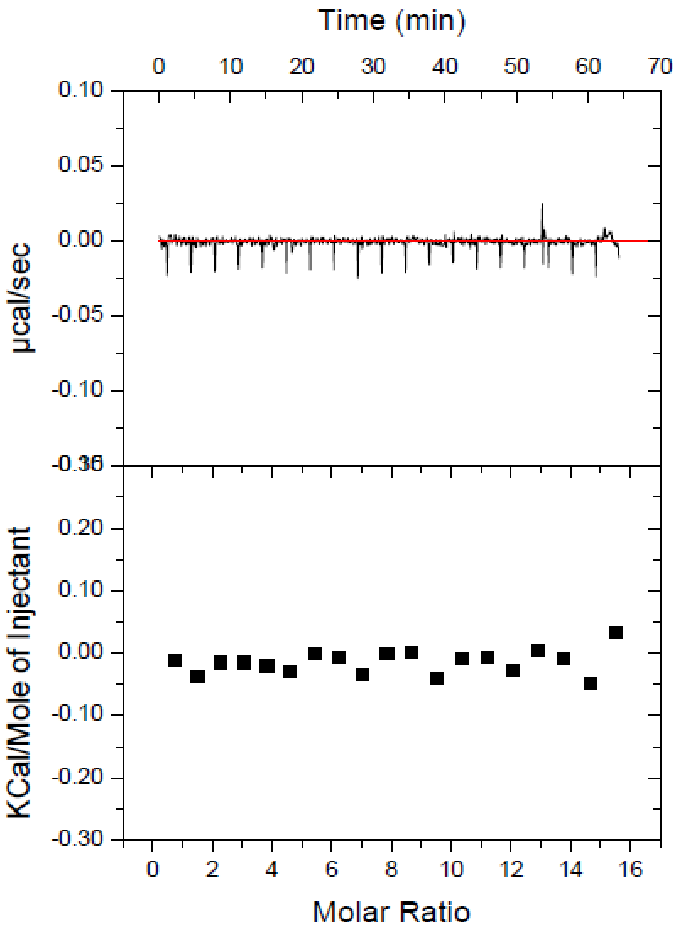

Supplementary Figure S1a. ITC isotherm of 20 m*M* Ca^{2+} titrated into 50 m*M* Na-HEPES pH 7.2.

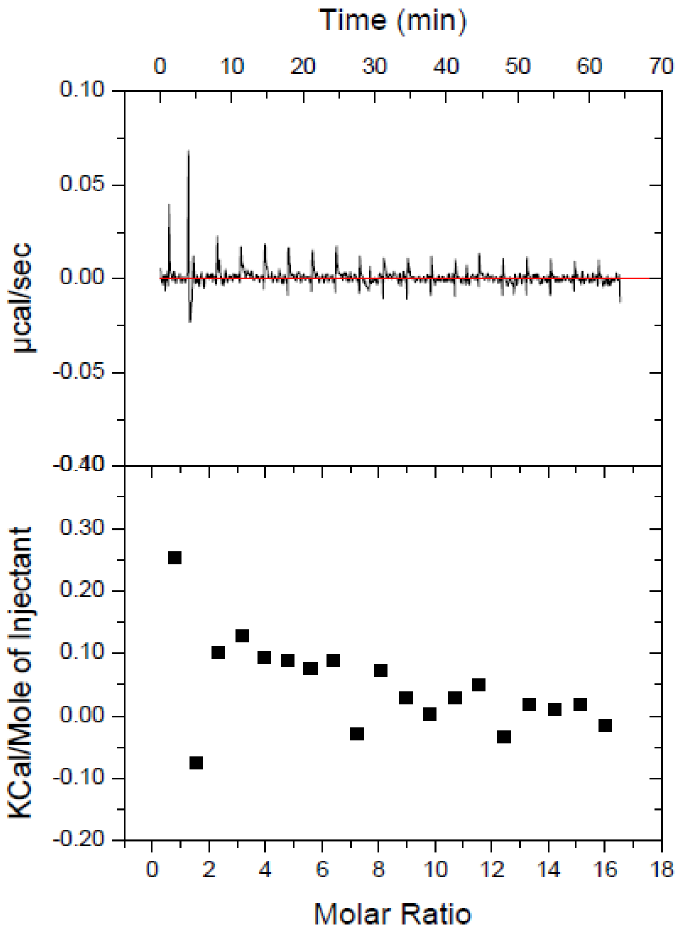

Supplementary Figure S1b. ITC isotherm of 20 m*M* Ca^{2+} titrated into 300 µ*M* LT3015 anti-LPA antibody in 50 m*M* Na-HEPES pH 7.2.

DNA sequences employed in production of naïve germline-encoded metalloantibody

>Ighv1-78
GAATTCATGGTGTCGGCTATCGTTCTCTACGTTCTCTTGGCAGCAGCGGCTCACTC
AGCGTTCGCCCAGGTTCAACTCCAGCAATCGGATGCGGAGCTGGTGAAGCCTGGA
GCTTCAGTCAAGATCTCCTGCAAGGTGAGCGGTTACACCTTCACTGACCACACCAT
CCACTGGATGAAGCAGCGCCCTGAGCAGGGCCTGGAATGGATCGGATACATCTAC
CCCCGTGACGGTTCCACTAAGTACAACGAGAAGTTCAAGGGCAAGGCTACCCTGA
CTGCCGACAAGTCCAGCTCTACCGCCTACATGCAGCTGAACTCCCTGACTAGCGA
AGACTCTGCTGTCTACTTCTGCGCCAGGGGTGGCTTCTACGGCAGCACCAACTAC
TTCGACTACTGGGGACAGGGTACCACTCTGACCGTCTCATCCGCTTCTACTAAGG
GACCATCAGTGTACCCACTGGCCCCTGGTTCCGCTGCTCAGACCAACAGCATGGT
CACTCTGGGTTGCCTGGTGAAGGGCTACTTCCCTGAGCCCGTGACCGTCACTTGG
AACTCTGGATCACTGAGCTCTGGTGTCCACACCTTCCCAGCTGTGCTGCAGTCTGA
CCTGTACACTCTGTCATCCAGCGTGACCGTCCCCTCTTCAACTTGGCCATCAGAAA
CCGTGACTTGCAACGTCGCTCACCCAGCCTCCAGCACCAAGGTGGACAAGAAGAT
CGTCCCTCGCGACTGCGGCCGCGGCGAAAACCTGTACTTCCAATCGGCGGGACA
TCATCACCACCACCACTAGAAGCTT

>Igkv17-121
GCTAGCATGGTCTCGGCAATCGTCCTCTACGTTCTCCTCGCAGCAGCAGCACACT
CGGCATTCGCGGAAACTACGGTTACGCAGTCCCCAGCGTCACTGTCCATGGCTAT
CGGCGAGAAAGTGACTATCCGCTGCATCACCTCCACTGACATCGACGACGACATG
AACTGGTACCAGCAGAAGCCAGGAGAGCCTCCCAAGCTGCTGATCTCTGAGGGTA
ACACCCTGCGCCCCGGCGTCCCATCACGTTTCTCCAGCTCTGGATACGGTACTGA
CTTCGTGTTCACCATCGAGAACATGCTGTCCGAAGACGTCGCCGACTACTACTGCC
TGCAGTCCGACAACCTGCCTCTGTTCACTTTCGGCAGCGGAACCAAGCTGGAGAT
CAAGAGGGCTGACGCTGCCCCCACTGTGAGCATCTTCCCACCTTCATCCGAACAG
CTGACCTCCGGTGGAGCCAGCGTGGTCTGCTTCCTGAACAACTTCTACCCTAAGG
ACATCAACGTGAAGTGGAAGATCGACGGATCTGAAAGGCAGAACGGTGTCCTGAA
CTCATGGACTGACCAGGACAGCAAGGACTCTACCTACTCAATGAGCTCTACCCTGA
CTCTGACCAAGGACGAGTACGAAAGACACAACTCTTACACTTGCGAAGCAACCCAT
AAGACCAGCACCTCACCTATTGTCAAAAGTTTCAACCGCAACGAATGTTAGGCATG
C

>Ighv1-78/Igkv17-121pFastBacDual
TTCTCTGTCACAGAATGAAAATTTTTCTGTCATCTCTTCGTTATTAATGTTTGTAATT
GACTGAATATCAACGCTTATTTGCAGCCTGAATGGCGAATGGACGCGCCCTGTA
GCGGCGCATTAAGCGCGGCGGGTGTGGTGGTTACGCGCAGCGTGACCGCTACAC
TTGCCAGCGCCCTAGCGCCCGCTCCTTTCGCTTTCTTCCCTTCCTTTCTCGCCACG
TTCGCCGGCTTTCCCCGTCAAGCTCTAAATCGGGGGCTCCCTTTAGGGTTCCGATT
TAGTGCTTTACGGCACCTCGACCCCAAAAAACTTGATTAGGGTGATGGTTCACGTA
GTGGGCCATCGCCCTGATAGACGGTTTTTCGCCCTTTGACGTTGGAGTCCACGTT
CTTTAATAGTGGACTCTTGTTCCAAACTGGAACAACACTCAACCCTATCTCGGTCTA
TTCTTTTGATTTATAAGGGATTTTGCCGATTTCGGCCTATTGGTTAAAAAATGAGCT
GATTTAACAAAAATTTAACGCGAATTTTAACAAAATATTAACGTTTACAATTTCAGGT
GGCACTTTTCGGGGAAATGTGCGCGGAACCCCTATTTGTTTATTTTTCTAAATACAT

TCAAATATGTATCCGCTCATGAGACAATAACCCTGATAAATGCTTCAATAATATTGA
AAAAGGAAGAGTATGAGTATTCAACATTTCCGTGTCGCCCTTATTCCCTTTTTTGCG
GCATTTTGCCTTCCTGTTTTTGCTCACCCAGAAACGCTGGTGAAAGTAAAAGATGC
TGAAGATCAGTTGGGTGCACGAGTGGGTTACATCGAACTGGATCTCAACAGCGGT
AAGATCCTTGAGAGTTTTCGCCCCGAAGAACGTTTTCCAATGATGAGCACTTTTAAA
GTTCTGCTATGTGGCGCGGTATTATCCCGTATTGACGCCGGGCAAGAGCAACTCG
GTCGCCGCATACACTATTCTCAGAATGACTTGGTTGAGTACTCACCAGTCACAGAA
AAGCATCTTACGGATGGCATGACAGTAAGAGAATTATGCAGTGCTGCCATAACCAT
GAGTGATAACACTGCGGCCAACTTACTTCTGACAACGATCGGAGGACCGAAGGAG
CTAACCGCTTTTTTGCACAACATGGGGGATCATGTAACTCGCCTTGATCGTTGGGA
ACCGGAGCTGAATGAAGCCATACCAAACGACGAGCGTGACACCACGATGCCTGTA
GCAATGGCAACAACGTTGCGCAAACTATTAACTGGCGAACTACTTACTCTAGCTTC
CCGGCAACAATTAATAGACTGGATGGAGGCGGATAAAGTTGCAGGACCACTTCTG
CGCTCGGCCCTTCCGGCTGGCTGGTTTATTGCTGATAAATCTGGAGCCGGTGAGC
GTGGGTCTCGCGGTATCATTGCAGCACTGGGGCCAGATGGTAAGCCCTCCCGTAT
CGTAGTTATCTACACGACGGGGAGTCAGGCAACTATGGATGAACGAAATAGACAG
ATCGCTGAGATAGGTGCCTCACTGATTAAGCATTGGTAACTGTCAGACCAAGTTTA
CTCATATATACTTTAGATTGATTTAAAACTTCATTTTTAATTTAAAAGGATCTAGGTG
AAGATCCTTTTTGATAATCTCATGACCAAAATCCCTTAACGTGAGTTTTCGTTCCAC
TGAGCGTCAGACCCCGTAGAAAAGATCAAAGGATCTTCTTGAGATCCTTTTTTTCTG
CGCGTAATCTGCTGCTTGCAAACAAAAAAACCACCGCTACCAGCGGTGGTTTGTTT
GCCGGATCAAGAGCTACCAACTCTTTTTCCGAAGGTAACTGGCTTCAGCAGAGCG
CAGATACCAAATACTGTCCTTCTAGTGTAGCCGTAGTTAGGCCACCACTTCAAGAA
CTCTGTAGCACCGCCTACATACCTCGCTCTGCTAATCCTGTTACCAGTGGCTGCTG
CCAGTGGCGATAAGTCGTGTCTTACCGGGTTGGACTCAAGACGATAGTTACCGGA
TAAGGCGCAGCGGTCGGGCTGAACGGGGGGTTCGTGCACACAGCCCAGCTTGGA
GCGAACGACCTACACCGAACTGAGATACCTACAGCGTGAGCATTGAGAAAGCGCC
ACGCTTCCCGAAGGGAGAAAGGCGGACAGGTATCCGGTAAGCGGCAGGGTCGGA
ACAGGAGAGCGCACGAGGGAGCTTCCAGGGGGAAACGCCTGGTATCTTTATAGTC
CTGTCGGGTTTCGCCACCTCTGACTTGAGCGTCGATTTTTGTGATGCTCGTCAGGG
GGGCGGAGCCTATGGAAAAACGCCAGCAACGCGGCCTTTTTACGGTTCCTGGCCT
TTTGCTGGCCTTTTGCTCACATGTTCTTTCCTGCGTTATCCCCTGATTCTGTGGATA
ACCGTATTACCGCCTTTGAGTGAGCTGATACCGCTCGCCGCAGCCGAACGACCGA
GCGCAGCGAGTCAGTGAGCGAGGAAGCGGAAGAGCGCCTGATGCGGTATTTTCT
CCTTACGCATCTGTGCGGTATTTCACACCGCAGACCAGCCGCGTAACCTGGCAAA
ATCGGTTACGGTTGAGTAATAAATGGATGCCCTGCGTAAGCGGGTGTGGGCGGAC
AATAAAGTCTTAAACTGAACAAAATAGATCTAAACTATGACAATAAAGTCTTAAACTA
GACAGAATAGTTGTAAACTGAAATCAGTCCAGTTATGCTGTGAAAAAGCATACTGG
ACTTTTGTTATGGCTAAAGCAAACTCTTCATTTTCTGAAGTGCAAATTGCCCGTCGT
ATTAAAGAGGGGCGTGGCCAAGGGCATGGTAAAGACTATATTCGCGGCGTTGTGA
CAATTTACCGAACAACTCCGCGGCCGGGAAGCCGATCTCGGCTTGAACGAATTGT
TAGGTGGCGGTACTTGGGTCGATATCAAAGTGCATCACTTCTTCCCGTATGCCCAA
CTTTGTATAGAGAGCCACTGCGGGATCGTCACCGTAATCTGCTTGCACGTAGATCA
CATAAGCACCAAGCGCGTTGGCCTCATGCTTGAGGAGATTGATGAGCGCGGTGGC
AATGCCCTGCCTCCGGTGCTCGCCGGAGACTGCGAGATCATAGATATAGATCTCA
CTACGCGGCTGCTCAAACCTGGGCAGAACGTAAGCCGCGAGAGCGCCAACAACC

GCTTCTTGGTCGAAGGCAGCAAGCGCGATGAATGTCTTACTACGGAGCAAGTTCC
CGAGGTAATCGGAGTCCGGCTGATGTTGGGAGTAGGTGGCTACGTCTCCGAACTC
ACGACCGAAAAGATCAAGAGCAGCCCGCATGGATTTGACTTGGTCAGGGCCGAGC
CTACATGTGCGAATGATGCCCATACTTGAGCCACCTAACTTTGTTTTAGGGCGACT
GCCCTGCTGCGTAACATCGTTGCTGCTGCGTAACATCGTTGCTGCTCCATAACATC
AAACATCGACCCACGGCGTAACGCGCTTGCTGCTTGGATGCCCGAGGCATAGACT
GTACAAAAAAACAGTCATAACAAGCCATGAAAACCGCCACTGCGCCGTTACCACCG
CTGCGTTCGGTCAAGGTTCTGGACCAGTTGCGTGAGCGCATACGCTACTTGCATTA
CAGTTTACGAACCGAACAGGCTTATGTCAACTGGGTTCGTGCCTTCATCCGTTTCC
ACGGTGTGCGTCACCCGGCAACCTTGGGCAGCAGCGAAGTCGAGGCATTTCTGTC
CTGGCTGGCGAACGAGCGCAAGGTTTCGGTCTCCACGCATCGTCAGGCATTGGC
GGCCTTGCTGTTCTTCTACGGCAAGGTGCTGTGCACGGATCTGCCCTGGCTTCAG
GAGATCGGTAGACCTCGGCCGTCGCGGCGCTTGCCGGTGGTGCTGACCCCGGAT
GAAGTGGTTCGCATCCTCGGTTTTCTGGAAGGCGAGCATCGTTTGTTCGCCCAGG
ACTCTAGCTATAGTTCTAGTGGTTGGCCTACGTACCCGTAGTGGCTATGGCAGGG
CTTGCCGCCCCGACGTTGGCTGCGAGCCCTGGGCCTTCACCCGAACTTGGGGGT
TGGGGTGGGGAAAAGGAAGAAACGCGGGCGTATTGGTCCCAATGGGGTCTCGGT
GGGGTATCGACAGAGTGCCAGCCCTGGGACCGAACCCCGCGTTTATGAACAAAC
GACCCAACACCCGTGCGTTTTATTCTGTCTTTTTATTGCCGTCATAGCGCGGGTTC
CTTCCGGTATTGTCTCCTTCCGTGTTTCAGTTAGCCTCCCCCATCTCCCGGTACCG
CATGCCTAACATTCGTTGCGGTTGAAACTTTTGACAATAGGTGAGGTGCTGGTCTT
ATGGGTTGCTTCGCAAGTGTAAGAGTTGTGTCTTTCGTACTCGTCCTTGGTCAGAG
TCAGGGTAGAGCTCATTGAGTAGGTAGAGTCCTTGCTGTCCTGGTCAGTCCATGA
GTTCAGGACACCGTTCTGCCTTTCAGATCCGTCGATCTTCCACTTCACGTTGATGT
CCTTAGGGTAGAAGTTGTTCAGGAAGCAGACCACGCTGGCTCCACCGGAGGTCAG
CTGTTCGGATGAAGGTGGGAAGATGCTCACAGTGGGGGCAGCGTCAGCCCTCTT
GATCTCCAGCTTGGTTCCGCTGCCGAAAGTGAACAGAGGCAGGTTGTCGGACTGC
AGGCAGTAGTAGTCGGCGACGTCTTCGGACAGCATGTTCTCGATGGTGAACACGA
AGTCAGTACCGTATCCAGAGCTGGAGAAACGTGATGGGACGCCGGGGCGCAGGG
TGTTACCCTCAGAGATCAGCAGCTTGGGAGGCTCTCCTGGCTTCTGCTGGTACCA
GTTCATGTCGTCGTCGATGTCAGTGGAGGTGATGCAGCGGATAGTCACTTTCTCG
CCGATAGCCATGGACAGTGACGCTGGGGACTGCGTAACCGTAGTTTCCGCGAATG
CCGAGTGTGCTGCTGCTGCGAGGAGAACGTAGAGGACGATTGCCGAGACCATGC
TAGCACCATGGCTCGAGATCCCGGGTGATCAAGTCTTCGTCGAGTGATTGTAAATA
AAATGTAATTTACAGTATAGTATTTTAATTAATATACAAATGATTTGATAATAATTCTT
ATTTAACTATAATATATTGTGTTGGGTTGAATTAAAGGTCCGTATACTCCGGAATATT
AATAGATCATGGAGATAATTAAAATGATAACCATCTCGCAAATAAATAAGTATTTTAC
TGTTTTCGTAACAGTTTTGTAATAAAAAAACCTATAAATATTCCGGATTATTCATACC
GTCCCACCATCGGGCGCGGATCCCGGTCCGAAGCGCGCGGAATTCATGGTGTCG
GCTATCGTTCTCTACGTTCTCTTGGCAGCAGCGGCTCACTCAGCGTTCGCCCAGG
TTCAACTCCAGCAATCGGATGCGGAGCTGGTGAAGCCTGGAGCTTCAGTCAAGAT
CTCCTGCAAGGTGAGCGGTTACACCTTCACTGACCACACCATCCACTGGATGAAG
CAGCGCCCTGAGCAGGGCCTGGAATGGATCGGATACATCTACCCCCGTGACGGTT
CCACTAAGTACAACGAGAAGTTCAAGGGCAAGGCTACCCTGACTGCCGACAAGTC
CAGCTCTACCGCCTACATGCAGCTGAACTCCCTGACTAGCGAAGACTCTGCTGTCT
ACTTCTGCGCCAGGGGTGGCTTCTACGGCAGCACCAACTACTTCGACTACTGGGG

ACAGGGTACCACTCTGACCGTCTCATCCGCTTCTACTAAGGGACCATCAGTGTACC
CACTGGCCCCTGGTTCCGCTGCTCAGACCAACAGCATGGTCACTCTGGGTTGCCT
GGTGAAGGGCTACTTCCCTGAGCCCGTGACCGTCACTTGGAACTCTGGATCACTG
AGCTCTGGTGTCCACACCTTCCCAGCTGTGCTGCAGTCTGACCTGTACACTCTGTC
ATCCAGCGTGACCGTCCCCTCTTCAACTTGGCCATCAGAAACCGTGACTTGCAAC
GTCGCTCACCCAGCCTCCAGCACCAAGGTGGACAAGAAGATCGTCCCTCGCGACT
GCGGCCGCGGCGAAAACCTGTACTTCCAATCGGCGGGACATCATCACCACCACCA
CTAGAAGCTTGTCGAGAAGTACTAGAGGATCATAATCAGCCATACCACATTTGTAG
AGGTTTTACTTGCTTTAAAAAACCTCCCACACCTCCCCCTGAACCTGAAACATAAAA
TGAATGCAATTGTTGTTGTTAACTTGTTTATTGCAGCTTATAATGGTTACAAATAAAG
CAATAGCATCACAAATTTCACAAATAAAGCATTTTTTTCACTGCATTCTAGTTGTGGT
TTGTCCAAACTCATCAATGTATCTTATCATGTCTGGATCTGATCACTGCTTGAGCCT
AGGAGATCCGAACCAGATAAGTGAAATCTAGTTCCAAACTATTTTGTCATTTTTAAT
TTTCGTATTAGCTTACGACGCTACACCCAGTTCCCATCTATTTTGTCACTCTTCCCT
AAATAATCCTTAAAAACTCCATTTCCACCCCTCCCAGTTCCCAACTATTTTGTCCGC
CCACAGCGGGGCATTTTTCTTCCTGTTATGTTTTTAATCAAACATCCTGCCAACTCC
ATGTGACAAACCGTCATCTTCGGCTACTTT
